# Active Inference and Social Actors: Towards a Neuro-Bio-Social Theory of Brains and Bodies in Their Worlds

**DOI:** 10.1007/s11577-024-00936-4

**Published:** 2024-03-19

**Authors:** Jacob E. Cheadle, K. J. Davidson-Turner, Bridget J. Goosby

**Affiliations:** 1 Department of Sociology, Population Research Center, and The Center on Aging and Population Sciences, The University of Texas at Austin, 305 E. 23rd St., 78712 Austin, TX USA; 2 The University of Texas at Austin, 305 E. 23rd St., 78712 Austin, TX USA

**Keywords:** Neurosociology, Biosociology, Brain, Predictive coding, Active inference, Neurosoziologie, Biosoziologie, Gehirn, Predictive Coding, Active Inference

## Abstract

Although research including biological concepts and variables has gained more prominence in sociology, progress assimilating the organ of experience, the brain, has been theoretically and technically challenging. Formal uptake and assimilation have thus been slow. Within psychology and neuroscience, the traditional brain, which has made brief appearances in sociological research, is a “bottom–up” processor in which sensory signals are passed up the neural hierarchy where they are eventually cognitively and emotionally processed, after which actions and responses are generated. In this paper, we introduce the Active Inference Framework (AIF), which casts the brain as a Bayesian “inference engine” that tests its “top–down” predictive models against “bottom–up” sensory error streams in its attempts to resolve uncertainty and make the world more predictable. After assembling and presenting key concepts in the AIF, we describe an integrated neuro-bio-social model that prioritizes the microsociological assertion that the scene of action is the situation, wherein brains enculturate. Through such social dynamics, enculturated brains share models of the world with one another, enabling collective realities that disclose the actions afforded in those times and places. We conclude by discussing this neuro-bio-social model within the context of exemplar sociological research areas, including the sociology of stress and health, the sociology of emotions, and cognitive cultural sociology, all areas where the brain has received some degree of recognition and incorporation. In each case, sociological insights that do not fit naturally with the traditional brain model emerge intuitively from the predictive AIF model, further underscoring the interconnections and interdependencies between these areas, while also providing a foundation for a probabilistic sociology.

## Abstract (de)

Trotz der zunehmenden Prominenz einer biologische Konzepte und Variablen einbeziehenden Forschung in der Soziologie steht ein ähnlicher Fortschritt bei der Berücksichtigung unseres Erfahrungsorgans, des Gehirns, in die soziologische Forschung vor theoretischen und technischen Herausforderungen. Eine formale Umsetzung und Integration ging daher bisher nur langsam voran. In der Psychologie und in den Neurowissenschaften wurde das Gehirn traditionell als ein „Bottom-up“-Prozessor angesehen, bei dem sensorische Signale entlang der neuronalen Hierarchie in höhere Hirnregionen weitergeleitet werden, wo sie schließlich kognitiv und emotional verarbeitet und woraufhin Aktionen und Reaktionen generiert werden. Diese traditionelle Sicht auf das Gehirn wurde in der soziologischen Forschung vereinzelt aufgegriffen. In diesem Beitrag stellen wir das Active Inference Framework (AIF) vor, demzufolge das Gehirn eine Bayesianische „Inferenzmaschine“ ist, die ihre „Top-down“-Vorhersagemodelle anhand von „Bottom-up“-Wahrnehmungen von Vorhersagefehlern prüft, um Unsicherheiten zu beseitigen und die Welt berechenbarer zu machen. Nach einer Zusammenstellung und Einführung von Schlüsselkonzepten des AIF beschreiben wir ein integriertes neuro-bio-soziales Modell, das der mikrosoziologischen These folgt, dass die Situation der Schauplatz des Handelns ist, in der Gehirne sich enkulturieren. Durch solche sozialen Dynamiken teilen enkulturierte Hirne Modelle der Welt miteinander und ermöglichen so kollektive Realitäten, die für die Zeit und den Ort angemessene Handlungen nahelegen. Abschließend wird dieses neuro-bio-soziale Modell im Kontext exemplarischer soziologischer Forschungsfelder diskutiert, darunter die Stress- und Gesundheitssoziologie, die Emotionssoziologie und die kognitive Kultursoziologie – alles Bereiche, in denen die Rolle des Gehirns in gewissem Maße beachtet und einbezogen wurde. In jedem Fall ergeben sich soziologische Erkenntnisse, die nicht in das traditionelle Hirnmodell passen, intuitiv aus dem prädiktiven AIF-Modell. Es verweist auf Verbindungen und Abhängigkeiten zwischen diesen Forschungsfeldern und bietet gleichzeitig eine Grundlage für eine probabilistische Soziologie.

## Introduction

This article proposes that incorporating insights from the cognitive and affective neurosciences will help to move sociology’s developing bio-social frameworks forward (e.g., Harris and McDade 2018; Ignatow 2021; Goosby et al. 2018), unlocking fresh avenues for producing comprehensive and impactful research across various subdomains within the social and human sciences. Additionally, by grounding its understanding of human actors with well-defined assumptions and principles (Friston 2009, 2010; Clark 2013), adopting a *neurosociological* perspective has the potential to foster more insightful and generative social analysis (Franks 2010, 2019; Kalkhoff et al. 2016). To the limited extent that “brains” make appearances within sociological research, we suggest that they tend to come along as hidden variables stowed away within assumptions about actors, their traces scattered across substantive areas to varying degrees according to the perceived theoretical needs of each area. The neural foundations of sociological actors thus incline toward the implicit and run the risk of being opaque, misleading, outdated, and/or wrong if not properly informed by some degree of interdisciplinary—if not transdisciplinary—engagement with contemporary neuroscience (Lizardo et al. 2020; Ignatow 2021). Because the neurosciences are currently engaged in their own theoretical audits and overhauls (Clark 2023), it is an opportune time to reevaluate both the neural principles of social actors and to consider where such understandings might fit within, contribute to, and reciprocally benefit from, sociological research.

Seen from this vantage point, the neurosociological endeavor holds the promise of advancing both biosocial theory and those facets of sociological theory in which actors are the central subjects or constituents. To these ends, we present an introduction to the *active inference framework *(AIF) from theoretical neuroscience (Friston 2013; Friston et al. 2017a), in which the brain is presented as a Bayesian “inference engine” whose computations comprise *probabilistic models of its body in its world* (Parr et al. 2022; Clark 2015; Barrett 2017a). This Bayesian perspective clarifies assumptions about brain dynamics and mechanisms within a principled normative account, elucidating how action and perception collaboratively minimize prediction errors through active engagement with the environment, thereby reciprocally optimizing its predictive models and guiding action (Parr et al. 2022). Importantly, the AIF view differs foundationally from traditional models of the brain within psychology and neuroscience, which have developed largely from “bottom–up”, stimulus response-driven conceptualizations (Barrett 2020; Barrett and Simmons 2015; Hutchinson and Barrett 2019). Where the traditional view emphasizes how actors *respond, react*, are *triggered,* or *activated *as experience is “processed,” the new paradigm accentuates *simulation, prediction, preparation, anticipation, prospection,* and *expectation* (Bubic et al. 2010).

Because this paper is programmatic and introductory, it emphasizes neuroscientific concepts that must be understood prior to rigorous sociological application. The goal here is thus to provide a basic understanding of the AIF from which the inclined can embark upon their own neurosociological investigations and biosocial research applications. To provide a degree of sociological orientation, we furnish thematic “breadcrumb trails” to example areas that we see benefitting from an updated and modernized AIF-informed understanding of the brain: stress and health, emotions, cognitive cultural sociology, and the notion of a probabilistic sociology (Strand and Lizardo 2022b). The paper is organized to converge upon these sociological themes within a unifying *neuro-bio-social* model. To these ends, we begin briefly with the traditional *bottom*–*up model* of the brain, followed by some (at least insinuated brains) in the sociological illustration areas. Next, we introduce *predictive coding* and the *Bayesian brain* concepts. Once established, we introduce the AIF, which adds a principled normative description of the roles of perception, action, decision making and planning, and learning within a socioenvironmental context. In the final section, once all the key concepts have been described, we present the conceptual model of a probabilistic sociological actor in terms of the neuro-bio-social interdependencies among the exemplar sociological topic areas.

## Overview of the Traditional Brain

Our goal is to present the predictive Bayesian brain and some of its implications via the AIF at a level of abstraction that provides some guidance to how a social scientist may think of social actors theoretically, but that does not necessitate use of the methods (e.g., fMRI) and materials (i.e., experimental paradigms) of neuroscientific research *per se*^1^. The physicalist perspective we emphasize considers “whole-brain” activity to be like an orchestra and the symphony it produces to be the conscious mind (see, for example, Seth 2021; Clark 2023; Hawkins and Dawkins 2021). Thus, it is important to recognize that by “the brain” we mean the organ, and by “the mind” we mean the consciousness that arises from its collective and integrated computational activity (Sandved-Smith et al. 2021; Seth 2021; Barrett and Satpute 2013; Barrett 2014). Because most neuroscience has sought knowledge generation at levels whose sociological relevance is usually not obvious (e.g., regional BOLD activation during an experimental task), the brain has been an indirect and peripheral *informant* in sociological inquiry^2^, especially when compared with the accessibility of the conscious mind to social science measurement modalities and the direct sentient experiences that foster social scientists’ intuitions and insights.

**Fig. 1:**
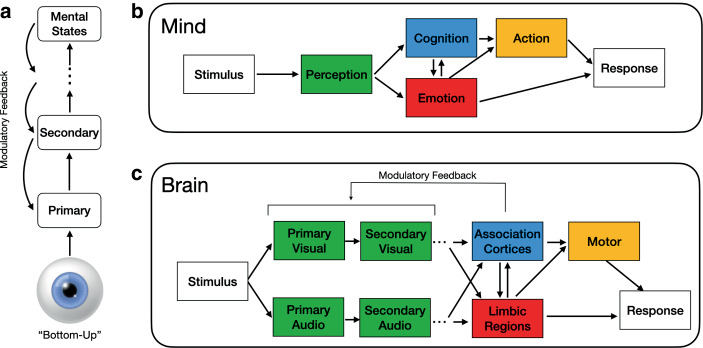
The “bottom–up” brain model. **a** Internal representations are generated by bottom–up input and top–down signals act as modulatory signals. Adapted from (Keller and Mrsic-Flogel 2018). **b** A mind-level depiction of the representational framework. **c** A brain-level representation of this framework. The colors characterize the mapping of mental faculties to their mapping in the brain (*green*: sensation and perception; *blue*: cognition; *red*: emotion; *yellow*: action). Adapted from Hutchinson and Barrett (2019)

The brain, as traditionally conceived since at least Sherrington (1900; in Keller and Mrsic-Flogel 2018, p. 424), is a “bottom–up” information filter and processor (Barbas 2015; Bubic et al. 2010), as depicted in Fig. 1a. Central to this model is the assumption that sensory information ascends “bottom–up” through the cortical hierarchy. During this journey, signals are thought to be processed via a series of filters and feature extractors, the results of which are compared with stored patterns acquired from prior experiences to facilitate understanding the environment and to guide behavior. This dynamic process supports perception, learning, and decision making, ultimately enabling more advanced cognitive and emotional processing, including the implementation of top–down control mechanisms. More detailed representations of the mind and brain are shown in Fig. 1b, c. The traditional brain is commonly conceived as being composed of specific and evolved (and possibly highly specialized) neural circuits, increasingly recognized as being webbed together into complex structural and function networks (Bassett and Sporns 2017; Farahani et al. 2019), where information is stored in the memory so that it can be retrieved for future deployment and modulation of bottom–up processes (Keller and Mrsic-Flogel 2018).

The Fig. 1 depictions express the common understanding about how stimuli are represented in the mind, a precondition for learning and internalizing social experiences. The “bottom–up” cascades are *retrospective* in the sense that the computational activity is triggered or instantiated by the stimulus. Neural activity is thus often described in the language of *response/reaction/triggering/activating*, terms that are unsurprisingly ubiquitous in research across disciplines that either implicitly or explicitly draw upon this or a relatedly hypothesized model of brain and mind. Notably, this view of the brain has been criticized for failing to develop an integrated theory (Hawkins and Dawkins 2021), relying instead upon processing strategies and computational heuristics that are often bespoke and tailor-made for particular sensory or cognitive contexts (Walsh et al. 2020). However, we propose that this traditional understanding of the brain has implications for several areas of sociological research, even though the natural tendency is to emphasize capacities and conscious experiences of the mind, rather than the underlying physical mechanisms and dynamics.

### A Sketch of Three Brains in Sociology

Prior to presenting the AIF model, we provide brief introductions to brains, or at least the suggestive outlines, in a trio of example areas in sociology. We do not have room to explore these examples in depth; rather, our goal is to provide brief overviews and to give some analytic targets that we will return to after motivating an integrated neuro-bio-social model. We present research in health, emotions, and cognitive cultural sociology, all areas that have either implied or implicit brains within their frameworks. The AIF model, in our view, naturally lends itself to these areas as they each speak to different aspects of the model we present later, once all the pieces are in place.

For example, the brain in *sociological stress process* research (Pearlin 1989; Pearlin et al. 1981) echoes a biopsychosocial framework to capture the ways in which social structures organize psychological and physical health processes (Aneshensel and Mitchell 2014). The *physiological stress response *(Sapolsky 2004) (i.e., limbic regions and motor preparation in Fig. 1) is proposed as the translational mechanism by which stress-related patterns of physiological regulation accumulate to undermine health (for a review see Guidi et al. 2021; see also McEwen 1998a, b; McEwen and Seeman 1999). This model commonly draws upon the hypothalamus–pituitary–adrenal (HPA) axis to “kickstart” physiological cascades following perception of a stressor (McEwen and Akil 2020 and Goosby et al. 2018 also discuss the Sympathetic–Adrenal–Medullary [SAM] axis), including responses to mental events, as with *anticipatory stress* (Sapolsky 2004; McEwen and Gianaros 2010). The notion of anticipatory stress is key to understanding how social conditions undermine health, yet the theoretical fit of this concept is awkwardly situated as a response to mental events. Below, we provide two examples of how the model we describe provides reconsideration of the stress response. First, by grounding the “stress response” as a special case within the broader purview of what the brain, as the primary control center and regulator of its body, does. Second, we re-evaluate anticipatory stress in a more natural, predictive framework in which stress regulation is a type of *action* that is structured and organized by social conditions (Pearlin 1989), providing a deeper integration of the central contribution sociologists have made to mental and physical health research: characterization of how social structures and concomitant patterns of social (i.e., statistical; see Link and Phelan 1995; Glass and McAtee 2006) regularities shape the distributions and temporal patterns of stressors, symptoms, and ameliorative factors (e.g., personal resources, social support).

The sociology of emotions is another area that incorporates biosocial and neurosociological ideas about brains, bodies, and social contexts (Turner 2007, 2020). In what follows, we consider a foundational issue that we believe remains unresolved in the sociology of emotions; namely, how to define them. Emotion research lacks a convergent definition of emotions as well as a cohesive and organizing framework bringing its insights together (for reviews, see Bericat 2016; Stets 2010, 2012; Thoits 1989; Turner 2009; Turner and Stets 2006, 2006). We suggest that any definition might need to integrate body, cognition, culture, and the social situations and settings in which experiences unfold (i.e., Collins 1981, 1993, 2005). Turner (2007, 2020) has gone the farthest (in all of sociology, in our view) in grappling with the complexities and general sociological relevance of the brain, with respect to both emotions and evolution. His work also emphasizes the brain’s bottom–up and responsive nature, positing the evolution of dedicated neural circuitry^3^ for a basic set of emotions and their higher-order elaborations^4^. Although this work makes a strong case for the importance of the evolutionary expansion of emotional capacities for the development of sociality, social coordination, and cognition, a predictive view will reframe and, in some ways, complicate, and in other ways simplify, how emotions are understood. To a certain extent, our divergence from Turner mirrors longstanding concerns about how biological versus social emotions are (see Turner 2009). We argue that such distinctions resolve in the model we present in the same way in which a coin is made up of two inseparable sides.

Cognitive cultural sociologists have incorporated a broad-scope model of the dynamics of a brain inspired by the idea of “thinking fast and slow” from behavioral cognitive psychology^5^ (Kahneman 2011). Such “dual processing” frameworks propose that cognition is batched into two broad processing streams, “Type 1” (slow learning, associative, automatic, effortless), and “Type 2” (fast learning, propositional, slow, deliberate, effortful) (Lizardo et al. 2016). In terms of Fig. 1, this amounts to how pathways are “activated” by whatever is taking place such that a stimulus might bypass cognitive/association cortices directly through limbic structures (Leschziner 2019), perhaps activating fast cognitions or schema (Boutyline and Soter 2021) by failing to trigger the bidirectional association-limbic pathways in Fig. 1. Enculturation within dual-process frameworks can be conceived in terms of learning, remembering, thinking, and acting phases for each of the Type 1 and Type 2 cognitive processes (Lizardo et al. 2016). The model we present below provides an integrated approach to these different factors, including proposing a model of action tied to learning, remembering, and thinking via the neural dynamics of action and perception. Cultural sociologists have also recognized that a human brain has capacities that do not fit well within the dynamics of the traditional brain, such as *simulating the future* (Tavory and Eliasoph 2013), or uncertainties about how fast and slow processes interact (Lizardo et al. 2020).

In what follows, we introduce a model of the brain that provides a more natural and coherent framework for these kinds of issues. This brain is grounded in its cytoarchitectural composition and modulatory dynamics, as well as its informational flows and organization via its structural and functional networks (e.g., Chanes and Barrett 2016; Ficco et al. 2021; Hutchinson and Barrett 2019; Kleckner et al. 2017; Walsh et al. 2020). The AIF also provides a structured normative framework for understanding biological and cognitive processes (Parr et al. 2022), which is likely to be of broader sociological interest than specific neuroscientific findings centered primarily on the brain itself.

## The Predictive Brain

**Fig. 2:**
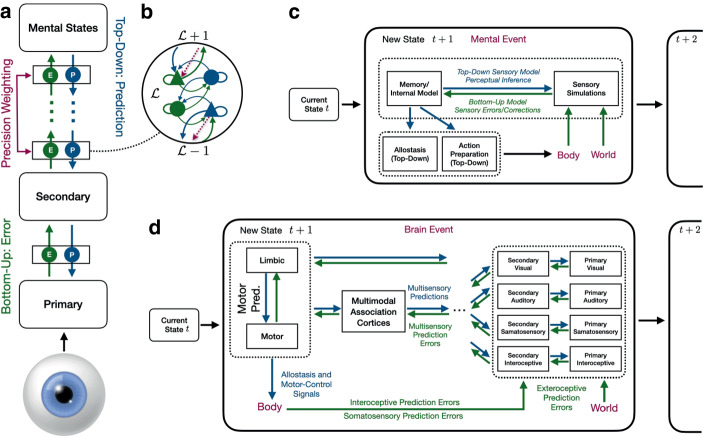
Predictive coding (i.e., “Bayesian Brain”) model. **a** In a hierarchical predictive processing framework, predictions cascade “top down” the hierarchy and are compared with errors ascending the hierarchy from the “bottom up”. Adapted from (Keller and Mrsic-Flogel 2018). The cutout for the neural dynamics is adapted from Seth (2013) and Friston and Kiebel (2009). **b** A mind-level depiction of the predictive processing framework with “top–down” and “bottom–up” informational flows. **c** A brain-level representation of this framework. Blue arrows represent predictions or hypotheses descending the computational hierarchy, whereas green arrows represent the sensory data that those predictions are compared against. Adapted from Hutchinson and Barrett (2019)

In this section we present an alternative model of the brain, the *hierarchical predictive coding* model (Rao and Ballard 1999), which, as shown in Fig. 2, reconceptualizes the traditional brain in Fig. 1 as functions of bidirectional informational streams, *E(rrors)* and *P(redictions)*. In this model, shown in terms of local state dynamics in Fig. 2a, brains simulate their sensory signals with top–down prediction (P) cascades^6^, which are compared with sensory information, providing a “bottom–up” error signal stream (E; compare with Fig. 1a) that rises up the hierarchy until it is explained (i.e., error signals do not need to propagate once explained by top–down signals). This (loosely) hierarchical^7^ predictive processing framework proposes a neural architecture that places inference as fundamental to information flow and integration throughout the brain. The supporting dynamics cover many scales of neuronal organization, interconnection, structure, and modulatory mechanisms (i.e., neurotransmitters, neuropeptides, and other molecules) (e.g., Barrett and Simmons 2015; Friston et al. 2017a; Hutchinson and Barrett 2019; Kleckner et al. 2017; Seth and Friston 2016).

An example neuronal architecture for a “state unit” is shown in the cutout in Fig. 2b, color coded for error (green) and prediction (blue) neurons, with projection neurons indicated as triangles and inhibitory interneurons indicated as circles (Friston and Kiebel 2009; Seth 2013). Top–down predictions flow from level ℒ + 1 to ℒ, whereas errors ascend from ℒ − 1 to ℒ so that prediction error within a state unit is a linear mixture of bottom–up and top–down connections. The upward flow of errors is precision (i.e., inverse variance) modulated by the dashed downward purple arrow (e.g., dopaminergic and oxytocin modulation; Seth and Friston 2016) so that errors viewed as imprecise can be downweighed and perhaps fail to propagate, signaling a need for enhanced attention to increase the precision of new sensory data (Friston 2009).

The idea is that brains attempt to encode and refine models that generate predictions by minimizing sensory errors and changing models (i.e., Hebbian plasticity, “neurons that fire together, wire together”) to better account for those errors that cannot be explained by experience under the current set of potential hypotheses or beliefs about the causes of sensory signals. When errors are adequately explained and predictions suffice, the top–down prediction is the signal. Top–down prediction streams are proposed to descend the computational hierarchy from areas of great compression and abstraction (i.e., the concept of a chair) and are decompressed in more granular representational areas whose collective activity assembles various features and details (e.g., the lines, edges, color, etc., of the chair). A key component of these dynamics is that neural circuits play dual roles, running predictions and processing sensory information about the states of its body and its world, including comparisons among different, competing models (i.e., Bayesian model averaging; see for example, Friston et al. 2017a; Hawkins and Dawkins 2021). Predictive processing thus reveals a unifying thread between perception, cognition, and imagination, pivotal elements of creativity, prospection, cognitive control, as well as psychopathological phenomena such as hallucinations (i.e., uncorrected prediction errors; Seth 2021; Barrett 2017a).

As shown in Fig. 2a for the mind and Fig. 2b for the brain, these inverse sensory streams include a combination of *exteroceptive* signals received from the environment and external to the body via the five senses, internal *interoceptive* signals received from the body about its large catalog of states (e.g., heart rate, glucose levels, hydration, sites of inflammation, etc.; Sterling 2012, 2015, 2020), and somatosensor*y* signals of body dynamics (e.g., body position and orientation [*proprioception*], touch). Figure 2 also emphasizes the ongoing and near-simultaneous interplay among multiple systems. For example, *allostasis* refers to the top–down predictive regulation of the internal milieu of the body (Sterling 2012), a systems concept that captures many signaling dynamics and physiological *actions* of the body on itself, situating body regulation as a set of coordinated activities across systems, organs, tissues, and so on. This concept extends well beyond the more traditional notion of reactive and system-specific homeostatic error-correcting feedback (Billman 2020), and has been conceptualized as comprising the processes that coordinate the contextual adjustments of expected homeostatic set-points (Pezzulo et al. 2015; Arnaldo et al. 2022). Brains anticipate all kinds of needs before they arise, whether it is raising blood pressure before standing, preparing the digestive system for an impending meal, signaling thirst before liquids are needed, or increasing available energy (i.e., adrenaline and cortisol secretion) when threatened (see Sterling 2012, 2015; Theriault et al. 2021).

Allostasis can therefore be understood as predictions about whether and how physiological states need to be coordinated or adjusted, including regulatory adaptations that seek to resolve prediction errors toward anticipated future states through (in)action. For example, low-energy states when sick (i.e., sickness behaviors; Shattuck and Muehlenbein 2015) and the metabolically costly “stress-response” states that energize “fight or flight” motor actions (Sterling 2012). A clear implication of allostatic thinking is that the accumulated “allostatic load” (McEwen 1998b) is not simply the result of the repeated activation of the physiological stress response or a pathologically locked-in response. It can also reflect the anticipatory preparation of the body for vicissitudes by making energy available (i.e., cortisol) and preparing the body for *potential* harm (e.g., wounds; Sapolsky 2004). Because *social structure* implies a degree of predictability and consistency (i.e., systemic racism; Bonilla-Silva 2015), Sterling (2012; see also Sterling and Platt 2022) advocates for system-level (i.e., sociological) changes to social and environmental conditions that alter the predictive landscape so that healthy regulatory patterns can be restored.

The “data” needed to regulate a body involve its various states, which are obtained via *interoception*, the catalog of signals ascending from throughout the body to the brain, including their interpretation and integration (Barrett and Simmons 2015; Chen et al. 2021; Kleckner et al. 2017). Interestingly for research on the sociology of emotions, interoception is proposed to be critical for emotional processes via the registration of affect in consciousness, which is thought to be an index, barometer, or summarizing filter for interoceptive signals (Barrett 2017a, 2020; Theriault et al. 2021), perhaps reflecting prediction error rates and their changes (Joffily and Coricelli 2013; Van de Cruys 2017). *Motor control* and other signaling pathways key to bodily action upon the world are also included in Fig. 2 in terms of predictions about the body’s physical dynamics (i.e., movement, action, and location), which are monitored via *proprioception*, a key part of the *somatosensory system* (e.g., touch). Here, it is worth recognizing that motor signal pathways support all physical action. For example, a highly social activity such as participating in a conversation is accomplished through sequences of precise motor commands that generate vocalizations, as well as all the other subtleties of facial, postural, and gestural expression. It is proprioception that makes individuals into *actors* who seek to exert control over their exteroceptive and interoceptive sensations through motor actions that affect the world, facilitating some control over regulatory allostatic demands and consequent cardiometabolic and immunological costs, and thus the body state feedbacks represented in consciousness as affect.

## The Active Inference Framework

One of the key challenges for the brain—if not *the* key challenge—is that it is in fact a *brain in the vat*^8^ (Gere 2004); it is hidden away inside the “black box” of the skull (Rieke et al. 1999, cited in Clark 2013). From this enclosed space, a brain makes *inferences* about the causes of the electrochemical and other signals it receives from inside (i.e., interoceptive), about (i.e., somatosensory), and outside of (i.e., exteroception) its body. Brains do not *see*, for example, they attempt to explain signals received from the eyes via the optic nerve, continuously correcting prediction errors and updating their representation of the environment, which is *experienced* as if *seeing* the world veridically^9^. Within the framework of the predictive brain, the prevailing context is *uncertainty *about body states, other conspecifics, and environmental features outward through the increasingly abstracted axis of nested hierarchies of social organization from the micro to the global (Bronfenbrenner 1977; Glass and McAtee 2006). What the inherent uncertainty outside of the brain’s vat entails of a predictive Bayesian brain is the core challenge that has been taken up within the *active inference framework* (AIF) (Friston 2009, 2010, 2013). Each brain in the AIF seeks to encode the statistical regularities of the *generative processes* of its embedding environments (i.e., culture, social structure) and body (i.e., its own capacities) in the *generative models* of its brain (i.e., models that generate probabilistic predictions defined as the joint probability distribution of observations and hypotheses/beliefs) (Bruineberg et al. 2018; Friston et al. 2017a; Parr et al. 2022; Ramstead et al. 2020; Smith et al. 2022).

It is worth pausing here on those two terms, *generative processes*, and *generative models*: much sociological research is explicitly dedicated to mapping the statistical regularities of the social world in terms of (for example) race, class, and gender, as well as their intersections (Grusky 2014; Grusky and Hill 2017). At the same time, core sociological interests in socialization and enculturation are fundamentally concerned with the beliefs about the world and the potential actions afforded^10^ by the sociocultural structures in which actors are embedded, and which are embodied in adaptive neural structures as predictive generative models. Indeed, the stress process, emotion, and cultural sociology each attend to different facets of the way that social structure “parameterizes” health, feeling, and different aspects of cognition in support of enculturation and action. The AIF provides a principled, normative framework for how it is that a predictive brain can become what it is and do what it can, namely be an organ of action in, and inference about, the generative processes comprising its embedding environments and structuring its experiences (Clark 2023; Parr et al. 2022).

### The Bayesian Brain

Our description of hierarchical predictive processing above and in Fig. 2 only tells part of the story of the predictive brain. In order to appreciate it more fully, it is important to understand why it is also sometimes referred to as a *Bayesian brain *(Friston 2010; Knill and Pouget 2004; Parr et al. 2022; Seth and Friston 2016). Many readers are probably at least somewhat familiar with Bayesianism by route of probability theory and Bayes’ Theorem^11^, and perhaps as an alternative to the “frequentism” that characterizes most contemporary statistical research (Clayton 2021). Of course, many others have likely spent their careers primarily within the frequentism that guides most contemporary quantitative research, or perhaps outside that framework almost entirely and with different epistemological commitments. One common distinction is that frequentism is based on “objective” probabilities estimated from data (i.e., a frequency divided by the number of samples), whereas Bayesian probability admits both objective and subjective probabilities (i.e., personal beliefs, judgments, or opinions about the likelihood of events occurring—or, for our purposes here, a brain’s beliefs about the causes of sensory events, such as the next word you will …). The distinctions generally do not matter for most contemporary statistical modeling, particularly those reliant on large samples assumed to have been collected at an approximately single point in time (i.e., wave). To an organism sampling a diverse array of experiences sequentially one after another through time, however, the distinction between these two approaches to probability is crucial.

Because we are all entrained in the unidirectional flow of time, the notion of model *updating* toward improved prediction error minimization is essential, and Bayes’ Theorem provides the optimal way to update conditional probabilities. The idea is that we have *prior* beliefs about the causes of sensations based upon previous experiences, which can be expressed as the probability distributions of hypotheses/beliefs about the states or causes of sensory experiences. These expectations are embodied across scales, in the state units of our neural circuits through neural hierarchies, in the collective dynamics that support our cognitions and memories, and throughout the evolutionary heritages of cells, organs, and other bodily mechanisms and systems (Kirchhoff et al. 2018; Ramstead et al. 2021; Sterling 2015, 2020). These priors also relate to *sampling probabilities,* termed the *likelihood*^12^ more generally*,* reflecting the probability distributions of obtaining certain sensory observations given our hypotheses/beliefs. Sampling probabilities thus quantify how well sensory inputs align with predictions, analogous to the frequentist notion of the parameters (i.e., hypotheses) that maximize the likelihood of observing some data.

What the predictive brain seeks to do is update prior beliefs into new *posterior *beliefs (i.e., *inferential probabilities*) that account for *new* observations so that predictions can be confirmed or improved going forward. Posterior beliefs are expressed as a probability distribution of hypotheses given observations (i.e., given what has now been observed, which hypothesis/belief is most probable?). This update involves a model inversion of the generative model, which is the joint distribution of hypotheses and observations. Via the multiplication rule^13^, the generative model can be expressed as the *sampling probabilities *(or *likelihood*) multiplied by the *priors*. This product is normalized over the marginal probability of the observations to produce the *posterior inferential probabilities*, which represent the beliefs about the causes of sensory experiences given what has been observed (Smith et al. 2022). The challenge is that the underlying causes of sensory experience are hidden behind the sensory veil enshrouding the brain^14^, so that inference is a model inversion of prior beliefs and observations given those beliefs, into beliefs given observations. That is, the translation goes from observed consequences given hypotheses (or beliefs) to inferring causes from their perceptual consequences (observations).

The model inversion provided by Bayes’ Theorem supplies the guide for how to update predictive generative models in a sequential way, experience by experience, over time, at various levels of the computational hierarchy. Predictions are thus priors and sensory experiences provide the observations. A high posterior probability of a hypothesis/belief in terms of a state computational unit (per Fig. 2b) is evidence supporting a model, in which case errors are not propagated back up the hierarchy (assuming that the prediction is also precise). Error propagation is thus a consequence of hypotheses or predictions at each step in the computational chain that are unlikely given what has been observed (possibly modulated by the precision of the belief). Such errors might have only local effects, such as updating a visual representation, or may have larger effects on generative models and thus subsequent cognitions and memory via long-term neuronal updating mechanisms (Friston et al. 2016; Parr et al. 2022). The idea is therefore that the brain models its exteroceptive, interoceptive, and somatosensory signals with generative models of the joint distributions of observations and hypotheses/beliefs about the causes of sensory signals, *but which are hidden and cannot be known directly because all the brain has access to is noisy signals received from its various sensors of the worlds inside and outside its body*.

### Variational Free Energy

**Fig. 3:**
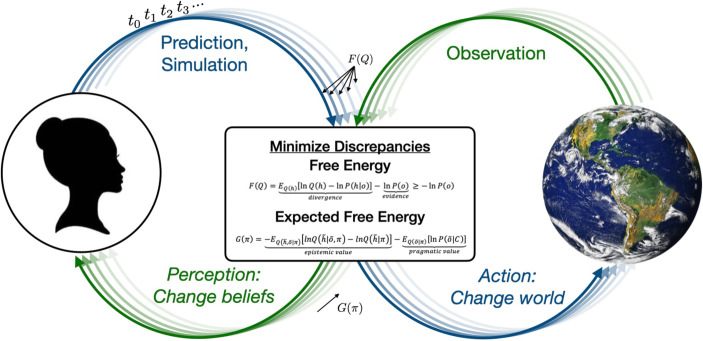
Action–perception cycles minimizing prediction–observation discrepancies in terms of current-state free energy and expected free energy minimization over time. Perception is used to test models through sensory observations whereas actions change the world, both by changing observations (i.e., a change in perspective) and by changing the world when it is directly acted upon. Adapted from Parr et al. (2022)

Brains seek to infer the most probable explanations for the states of their internal and external environments, which the AIF proposes is accomplished by Bayesian updating to optimize error minimizing generative models through temporal *action–perception cycle* sequences (Badcock et al. 2019; Parr et al. 2022). The idea is that *action* is the means by which actors “probe” the statistical regularities of the generative processes of their embedding environments, changing perspectives and perception, and it is *perception* that provides the observations by which generative models are tuned through the state dynamics of top–down prediction and bottom–up error cascades. Action facilitates learning (posterior update) or validates what has been learned (affirming priors; self-evidencing Hohwy 2016), whether by directly altering the external environment or by repositioning the actor within it to shift their perspective, thereby confirming or potentially altering beliefs. Inference is thus posed as a process of active engagement with the environment (see Ramstead et al. 2020). One way of depicting this model of action–perception cycles is shown in Fig. 3.

However, there is a subtle problem with the Bayesian model inversion as told so far. Bayesian inversion from sampling probabilities (i.e., likelihood) to posterior inferential probabilities often cannot be calculated because the probability distribution of observations (in the denominator), which is defined over the marginal probabilities of all possible observations over all possible hypotheses or states in the generative model, is unknown (Smith et al. 2022). Rather than a full Bayesian inversion, the AIF proposes converting the difficult challenge of *model inversion* into a much simpler *optimization* problem (Friston 2009, 2010, 2013) via *variational *(or approximate) Bayesian inference, drawing upon statistical models of predicted observations (Ramstead et al. 2020). To achieve this, a tractable variational approximation of the posterior is introduced as a distribution of states that is iteratively updated (i.e., gradient descent) to match the true posterior distribution achieved by exact inference as closely as possible, reflecting a neural implementation (i.e., Hebbian embodiment). This *recognition *or *variational density *is a “best-guess” Bayesian belief about the most likely causes of sensory observations. This best guess is optimized by minimizing a quantity called *variational free energy*, defined as a (Kullback–Liebler) divergence between this density and the true generative mode^15^.

With these changes, the difficult problem of Bayesian inverse inference becomes the more tractable optimization problem of variational free-energy minimization. One way to express this approach to free-energy minimization is shown in Fig. 3. The first term denotes the *divergence* between the approximate recognition and exact posterior distributions. This divergence term speaks to the role of perceptual inference, which is minimized as the recognition density better approximates the exact (unknown) posterior. In other words, perceptual inference minimizes free energy when the approximate posterior recognition density matches the posterior that would be obtained if it was possible to perform exact rather than variational Bayesian inference. In other words, perception optimizes free energy by confirming or changing generative models, and therefore predictions to minimize the divergence by revising beliefs or holding to those that are accurate. Importantly, this can amount to learning, and it is critical to the processes and dynamics by which individuals come to know what they know and expect what they do, speaking directly to cognition, emotion, socialization, and enculturation more broadly.

The second term is the negative logarithm of the probability of the observations (the value is large when the probability is small and approaches 0 as the probability goes to 1), an information theoretic quantity known as *surprisal* (Smith et al. 2022). When the divergence between the variational recognition density and the true posterior is minimized so that the model approximates the true posterior in the first model, free energy becomes an approximation to *surprisal*. Consequently, free energy is an upper bound on this quantity (Friston 2009, 2010), which Bayesian analysists know as negative log model evidence. What this means is that surprisal can be optimized *by changing sensory data* so that observations more closely resemble the model evidence (or marginal probability of the observations) to minimize surprisal. *This is achieved by engaging in actions that change observations to minimize prediction errors*. Because variational free energy quantifies the differences between expectations given prior beliefs and the observations our actions solicit, it represents prediction errors, *providing predictive processing with the look and feel of Bayesian inference* (Ramstead et al. 2020; Parr et al. 2022).

Consequently, the AIF motivates both perception and embodied action as two sides of inference that, when accomplished, minimize prediction errors to avoid surprising states, such as an unfortunate fish out of water—or as individuals of stigmatized social groups may feel when experiencing the threatening social exclusion of discriminatory experiences (Goosby et al. 2018), for example. There is a subtlety to this notion of surprisal because it may be tempting to think it of primarily in cognitive terms. We may predict an experience in advance so that surprisal at abstract cognitive levels is low, but may not intrinsically expect it at other levels of physical organization (Joffily and Coricelli 2013). A person riding a bike may recognize that a crash is imminent, avoiding a degree of surprise at an abstract cognitive level, whereas the rest of the body will soon be awash in surprisal at the physical trauma (which will consume the mind next through nociceptive somatosensory inference [i.e., pain]). Surprisal can thus index both cognitive expectations of the kinds with which cognitive cultural sociology is concerned, and inimical states that are profoundly unexpected at “lower” levels of computational hierarchies and physiological organization consistent with many concerns in health research. A person of color may thus be able to anticipate both potential discriminatory acts reflecting the racialized culture of their embedding environment and the way in which this environment directs the behavior of their conspecifics (Williams 2020), and still suffer the physiological allostatic consequences of “fight or flight” regulation that are naturally martialed given the social threats such exclusions imply (Cheadle et al. 2020; Jelsma et al. 2021).

### Expected Free Energy

Although variational free energy is a function of both the past and the present through the shaping of generative models, it is not *deeply prospective* in that it does not capture simulations about future observations and causes beyond current and next states (i.e., it is limited to present and past in predicting what is now and next; Friston et al. 2017a). In this sense, it is allostatic and anticipatory, but limited in that it only asks what is needed *now* to understand and/or change states to those that are anticipated *next *in a Markovian sequence^16^ (Friston et al. 2018). Indeed, although these are the very dynamics that are critical for understanding momentary acute stress regulation and concurrent affective dynamics, thus supporting the biological side of emotion states (Barrett 2017b, a; Joffily and Coricelli 2013; Van de Cruys 2017), this temporal bounding does not reflect the broader range of human capacities. Stress is not only acute, and chronic anticipatory stress is not merely a repetitive time series of acute stress responses to mental events, but rather stress can be predictive of both inevitability and uncertainty—*(un)certainties*—over different time scales. In other words, the future can happen *now* in our bodies, so we are prepared for it as it comes, even if we must wait for it. Beyond this, our inner worlds are often deeply cognitively engaged in our predictive capacities, simulating future and past events and encounters, forecasting contingences, and making plans for what we should do and how we should go about it (e.g., Schacter et al. 2012, 2007; Suddendorf 2013; Tavory and Eliasoph 2013).

*Expected free energy*, also depicted in Fig. 3, extends the AIF prospectively and arbitrarily forward in time (Friston et al. 2017a; Parr et al. 2022). To expand the time domain, *action policies*^17^ are introduced to include the kinds of thinking and planning (i.e., cognition) that are of broad sociological concern. In this case, expected free energy is managed over a sequence or trajectory of hidden states or hypotheses arbitrarily *into the future*, reframing variational free energy in terms of *expected states* and *expected observations* given *action policies*. An action policy is a set of hypotheses/beliefs about ways of acting and regulating the body, with the implication that actors capable of minimizing expected free energy treat planning and decision-making as a process of inferring what to do to achieve valued ends. Generative models parameterize these simulations and imaginings of potential futures, by which actors consider what results they hope to achieve, to consider what more they might need to know to realize these goals, and what action sequences may be enacted to those ends. Action plans are of course dynamic and are updated as new data are acquired, new opportunities emerge, priorities change, and so on, so expected free energy is scored for each potential action sequence to enable decision making and facilitate goal-directed action.

One way of expressing expected free energy, also notated in Fig. 3, is in terms of the sum of the *epistemic value* (or *information gain)* and *pragmatic value* (*expected utility* or *preference for specific observations*) of (in)action sequences^18^. Epistemic value is a negative expected divergence between the posterior and prior recognition densities. Because the term is negative, *maximizing *the difference of these two distributions minimizes expected free energy. The first distribution conditions on projected observations whereas the other does not, unlocking the value of actions that facilitate the acquisition of new information about the world useful for one’s own ends (which may, of course, prioritize others). The second term, pragmatic value (i.e., expected utility; Parr et al. 2022), captures preferences for particular observations, representing actions toward achieving the experiences one would like to have, over scales from what our cells want (e.g., glucose) to what our minds desire (e.g., acclaim). Action policies are thus sequences of epistemic and pragmatic actions that work to gather information about how to achieve desired goals and ends and then to go about realizing them.

Expected free energy therefore represents *best guesses* based upon hypothesized sequences of simulated outcomes from models entrained in prior experiences. There is no guarantee that an action plan is good and likely to succeed or that a good plan that is likely to succeed will be successful. Expected free energy also allows for risk-taking as it is a projected time-average variational free-energy minimization that may necessitate a willingness to tolerate short-run free-energy risk (i.e., gambling). Note too that expected free-energy minimization is fundamentally about conceiving actions that facilitate novelty and preferences. Expected free-energy minimization spotlights humans’ cognitive and emotional capabilities, including thinking, planning, coordinating with others, and enculturating these capacities so that individuals can participate in social systems with both distributed and shared resources. Consequently, the capacities supporting expected free-energy minimization within embedding social situational contexts enables some degree of control over variational free energy and thus metabolic and other resources. Those action policies that fail or cannot be realized are likely to increase free energy (i.e., prediction errors), requiring costly metabolic allostatic regulation patterns (Bobba-Alves et al. 2022; Arnaldo et al. 2022; Hutchinson and Barrett 2019; Theriault et al. 2021), pointing to the critical role of social and cultural structures in shaping opportunities and constraints (Pearlin 1989; Pearlin et al. 1981; Wheaton 2010), and by route of these, health and happiness (Sterling 2012, 2020). Because expected free energy accounts for both cognitive and physiological preferences for certain observations or outcomes, it clarifies the importance of social conditions in the link between what we actually experience versus what we hope to experience.

So much sociology is dedicated to how social conditions limit, block, undermine, and disenfranchise some groups relative to others that it is in many ways the science of barriers, impediments, restrictions, and constraints on potential action policies and thus on expected free-energy minimization. What happens in the flow of real life when expected free-energy minimization is undermined, moment by moment, is represented by variational free energy and its constituent metabolic and other costs. Here it may be useful to consider Maslow’s “Hierarchy of Needs” (Maslow 1964). This hierarchy begins with the evolved physiological requirement of a human body, followed by safety, love and belonging, esteem, cognitive needs, self-actualization, and transcendence. When viewed through the lens of the AIF, Maslow’s Hierarchy can be reinterpreted as a hierarchy of prediction error minimization and regulation, and so of free energy and expected free energy. Maslow’s lower physical needs are met when variational free energy is consistently well-optimized, and higher needs are met when action policies successfully regulate expected free energy and the realization of desired personally relevant and socially interdependent outcomes. Viewed in this way, the regulation and management of prediction errors speaks to canonical sociological questions about the costs and consequences that accrue for social groups who are statistically less likely to have their needs met when compared with those for whom such achievements are more likely. In other words, sociological insights about power, wealth, and privilege are recovered in the AIF in terms of basic biological and psychosocial imperatives (Parr et al. 2022).

### A Combined Neuro-Bio-Social Model

**Fig. 4:**
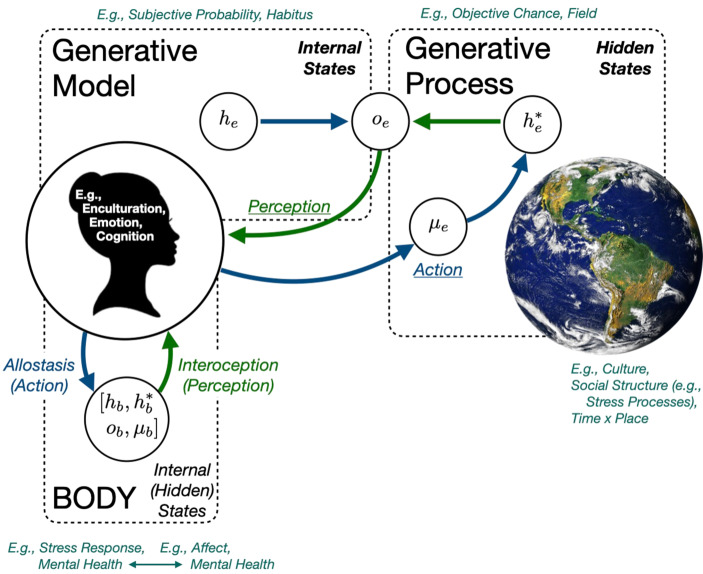
The combined Neuro-Bio-Social Model with select sociologically relevant annotations. The view presented is in reference to brain-mediated action-perception cycle time-dynamics both inside and outside of the body. Direct connections from the world to the brain/body (e.g., environmental toxins, viruses, etc.) are omitted (Goosby and Cheadle, this issue). Adapted from Parr et al. (2022)

With the pieces of the AIF now on the board, Fig. 4 arranges them. This figure explicitly represents the neuro-bio-social-*ness* of becoming—of *being*—a *person *of a particular time and place. What Fig. 4 emphasizes are the temporally interdependent dynamics among levels of social and biological organization from the actor’s perspective. This representation shifts from explicitly level-oriented arrangements of nested hierarchies of social and biological organization while allowing for the preservation of such distinctions as both causal and constitutive (e.g., Ylikoski 2013; see also Kirchhoff and Kiverstein 2019 for a diachronic perspective). For a human organism, Fig. 4 safeguards the microsociological assertion that the *scene of action* is the *situation *(Collins 1981, 1993, 2005; see also Boyns and Luery 2015) wherein actors learn about *generative processes* and develop their own *generative models* of them (Veissière et al. 2020). The generative process–model interplay is thus a fundamentally social project. This project, at least with regard to human actors, is intrinsically sociological, which provides the culturally situated and socially structured *settings* organizing the experiential histories that entrain generative models, laying down the paths that minds traverse on their situated journeys through time.

An important consequence is that actors’ *intra*subjective brains come to embody states learned through the situated experiences shared both directly and indirectly with other brains, becoming *inter*subjective by virtue of this and other culturally scripted experiential autocorrelations (e.g., Atzil et al. 2018; Bolis and Schilbach 2020; Fotopoulou and Tsakiris 2017; Misaki et al. 2021; Saxbe et al. 2020; Stephens et al. 2010). Through these intercorrelating social dynamics, actors learn by, and learn to, “think through other minds” by participating in collective regimes of attention (Veissière et al. 2020), enabling shared realities, expectations, communication, and cognitions as key features of generative models that are reproduced in each brain. Because actors actively contribute to one another’s generative models, this process of intersubjective autocorrelation becomes a catalyst for the social cohesions, identities, and solidarities that emerge from patterns of social situations, concatenating into, transforming, challenging, and dismantling social structures (Collins 1981, 1993, 2005). The collectivity of this activity makes the world more predictable in many ways, speaking to the inherent challenge of the epistemic uncertainty about the true but hidden states of, and their underlying causes in, the world. Along these lines, selected annotations are shown at the top of Fig. 4 to draw attention to important sociological conceptualizations that anticipated key features of uncertainty reducing generative process-model dynamics.

According to a pair of recent papers by Strand and Lizardo (2022a, b), Weber’s later works, which were not included in the original canon^19^ (Weber 1913, 2019), theorized distinctions between the *subjective probabilities* that individuals develop through experience to approximate the real and ultimately unknowable objective probabilistic states and chances of events in the world^20^ (i.e., *Chance*). This subjective–objective conceptualization, they suggest, influenced Bourdieu’s (e.g., Bourdieu 1973) later work where *field* came to capture distributions of objective *Chance* with its subjective approximation in the *habitus*. In fact, Strand and Lizardo (2022b) were inspired to call for a “probabilistic sociology” based on this reading of Weber and Bourdieu. We concur and propose a probabilistic sociology firmly rooted in well-established Bayesian principles in terms of generative processes and generative models, mirroring the Chance/field and subjective probability/habitus conceptualizations, with active inference providing the reconciling synthesis. Certainly, much sociological research is dedicated to identifying, parameterizing, and theorizing many such statistical, structural, and organizing features (e.g., Grusky 2014; Grusky and Hill 2017), represented as the *hidden states *(${h}_{e}^{*}$) of the generative processes of the world in Fig. 4. Not all hidden states are relevant to all actors and each brain attempts to learn about those statistically relevant as inferred from the signal patterns received through its sensorium (*o*_*e*_) (i.e., Hoffman 2019; Prakash et al. 2021). Each actor is thus constituted by the subsets of hidden states realized in the *internal states* (*h*_*e*_) of the brain’s generative models^21^ via neuronal updating and other modulatory mechanisms (i.e., predictive coding).

Actors seek to resolve or at least manage the inherent probabilistic uncertainties of the world outside through the local temporal dynamics of action–perception cycles, and over longer timespans via action policies entrained to the cultural affordances available to entities like them. Following Fig. 3, the arrows in Fig. 4 provide the dynamics of perception (*o*_*e*_) and action (*μ*_*e*_) cycles. Action alters perceptions by directly modifying the world in some way^22^, and/or by providing an alternative perspective from which new observations can be acquired. The consequences of action subserve the mechanisms of error (or *surprisal*) minimization via joint action-perception dynamics, by which it is proposed that actors (attempt to) maintain their preferred states over physiological$\leftrightarrow$psychological levels from the bodily and subconscious to the conscious (e.g., Maslow’s Hierarchy of Needs). Experience unfolds within the multifaceted sociological and environmental realities that shape how actors understand the distributions of their potential actions, the potential outcomes, and ultimately to explain the realized consequences. Figure 4 thus contains the implication, in the direct link between internal states and observed consequences ($h_{e}\rightarrow o_{e}$), that what is observed is dependent upon priors (i.e., generative models), and thus on the previous perceptual experiences in (or vicariously about) statistically similar settings, by which those prior internal states came to be embodied.

## Discussion

The embodied view of the predictive brain invites a return to sociological insights into the inherent uncertainty in the world and the need for humans to act in order to learn and make accurate inferences about it. Earlier, we suggested that brains have made some degree of appearance in sociological research, based largely on the traditional “bottom–up” model that is inherently retrospective. We propose that one consequence of this model is a reliance on a limited conception of the stress “response,” which is asked to do a lot of heavy lifting theoretically; that emotion research lacks a common definition of emotions while underappreciating affect; and that the model proposed here may provide the foundation for a theory of enculturation that ties together learning, remembering, and thinking via the neural dynamics of action and perception. Returning to these themes, Fig. 4 includes some clues into how the AIF informs and is informed by these three thematic “bread-crumb trails” by route of the centrality of the brain’s core predictive capacities and mechanisms. Moreover, Fig. 4 also gives rise to a kind of holism that emerges from the interdependencies among the dynamics of prediction, action, and perception, suggesting that to talk about one of these areas of sociological inquiry might often implicitly invoke the others.

Regarding mental and physical health and the concern for “how stress gets under the skin,” the core dynamic falls under the auspices of *allostasis* in this model and involves the coordinated resource allocation and consequent adjustment of homeostatic priors. The brain continuously monitors the states of the body and then attempts to adjust and regulate states for predicted situational demands. Successful prediction of the environment and successful physiological regulation together minimize variational free energy at lower levels of physiological mechanisms, processes, and needs, so that metabolic and other resources can be deployed *efficiently*. Of course, exteroceptive environmental prediction errors are frequently made and are sometimes threatening, posing risks to body (i.e., violence) and/or body–mind (i.e., interpersonal discrimination), giving rise to the concept of *acute stress*. Usually conceived of as *responses* to the (i.e., stimulus-response) recognition of threat or danger, such regulatory dynamics can also be viewed as (possibly evolutionarily selected, genetically encoded, and bodily realized) *predictions* about how to get ahead of the situation *now* by attempting to exert some degree of control over what happens *next*. Human actors accomplish *next* by releasing energic resources—writing a blank check to the body budget account as it were—to enable immediate and short-term “fight or flight” *action* (policies). The intent is to return perceptual states, whether physiologically vital to survival or in terms of conscious awareness, to those that are preferred.

The level-spanning nature of this from low-level physiological needs to mental abstractions brings forward the *stressor* as a concept that includes tremendous diversity over types, including duration, severity, level, and life-course timing (Aneshensel and Mitchell 2014; Wheaton 1994). Anticipating this, Sapolsky (2004) emphasized *anticipatory stress *as the scourge of the modern era. The idea he put forward is that our bodies only have the one stress response, whether or not a stressor is acute or anticipated, with the latter instantiated as a response to mental events. We suggest a subtler distinction: the mechanisms and bodily processes whose joint actions are called a *stress response* are not so special. The stress response is but a few discordant chords played upon the strings of cardiometabolic and related mechanisms by which the body is allostatically regulated *every moment of life*. Anticipatory stress as a response to mental events thus obfuscates as much as it illuminates. Instead, we propose considering it in terms of the anticipated dis-preferred states that result from epistemic *(un)certainties*, by which we mean both probabilistically *uncertain* and probabilistically *certain*. Such (un)certainties undermine actors’ abilities to manage their expected and free energy, leaving them to pay the local metabolic and other costs that come with higher regulatory free-energy bills (Bobba-Alves et al. 2022).

Anticipatory stress can thus alternatively be seen as a balancing act of *predicted demand* and *inherent (un)certainty *about what is coming in life. Sociological concerns about social structure and stress processes implicate generative processes in which individuals and groups of actors are *predictably* unable to enact action policies for preferences, or at least complete them after starting, blocking epistemic and pragmatic goal attainments. Actors in adverse conditions must act within the constraints on what they can learn toward what can and cannot be achieved, along with the *proliferating uncertainties* about what the costs of failures *could* be (i.e., stress proliferation; Pearlin et al. 1997). It seems likely, in fact, that much of what is chronic about modern stress reflects the jointness of the limitations on the scope of feasible action policies and the myriad uncertain follow-up consequences that arise when things go wrong. Disadvantage is often a lack of robustness to even small changes in already dis-preferred conditions. This jointness can confound selection of even less-preferred but potentially realizable action policies, increasing anxiety (Barrett 2017a), and thus the inference that the world is an innately dangerous place, abound with risk, that the body must accordingly be prepared for (Sterling 2012, 2018; Schulkin and Sterling 2019). The brain seeks to match its bodily energetics to its contexts as efficiently as possible, bringing along the broad packages of sociological stress processes that accumulate as functions of time^23^ (i.e., allostatic load and overload; Bobba-Alves et al. 2022).

Body regulation is central to the embodied and embodying phenomenology of experience beyond the physiological demands of stress. *Affect *in Fig. 4 provides the low-dimensional indexing of interoceptive monitoring of allostatic regulatory states and changes in terms of *valence* and *arousal* (and sometimes *dominance*; Russell 1980; Mehrabian 1980), and acute stress typically occupies a negative, energetic location within the affective-state space (negative valence, high arousal, and high dominance or lack of control). As von Scheve (2018) notes, affect is under-theorized in sociological research, but it plays a foundational role here by providing a mind-accessible summary index of how the brain understands its body states by route of its moment-to-moment feelings (Damasio 1999; Barrett 2017b; Duncan and Barrett 2007; Kleckner et al. 2017; Wormwood et al. 2019). In other words, affect signifies bodily states and their dynamic shifts, facilitating context-specific (i.e., situated) embodied inferences (Seth and Friston 2016; Barrett 2017a). Recent models, such as Barrett’s (2014, 2017a, b), proposes that emotions are *concepts*^24^ used to interpret and make sense of affective experiences against the backdrop of prediction error resolution rates within specific situational contexts (see also Joffily and Coricelli 2013; Van de Cruys 2017). That is, emotions categorize what we feel within the situation we are in *vis a vis* our predictions and are thus *inferences* about the joint distribution of causes in the body and the world.

Affect is thus posed as a low-dimensional summary of current and changing body states, whereas emotions *make meaning* of these feelings, elaborating them, situating them within their broader statistical panorama, and dimensionalizing them over the diverse conceptual terrain in which actors are enculturated. Emotions are therefore sociocultural constructs, grounded in biology, that make meaning from intero- and exteroceptive inputs through conceptual acts and guided by prediction error resolution (Barrett 2014, 2017a, b; Joffily and Coricelli 2013; Van de Cruys 2017; Van de Cruys and Wagemans 2011). Each emotion instance is proposed to be a realization of a “population of instances” over the unique features of situations, body state, and neural state dynamics at that time (Siegel et al. 2018), and are therefore posed as integrative and multimodal (i.e., relying on many different sources). This view challenges specific circuit-based proposals (not that there are not well-documented regions/circuits, hubs, and networks involved; Barrett 2017b) based in part on the failure to identify the “fingerprints” of specific emotions in the brain (Barrett and Satpute 2013; Lindquist et al. 2012; Wager et al. 2015). Consequently, and harkening back to both social constructionist and biological debates about such things (see Turner 2009; Turner and Stets 2006), *neither provides an accurate description without the other*. Emotions are in this way a duality like a coin: their existence emerges as the joining of both sides.

It is our proposal that this view provides a promising definitional grounding that could help to anchor a sociology of emotions in which there are nearly as many definitions as contributors (for reviews see Bericat 2016; Olson et al. 2017; Turner 2009; Turner and Stets 2006). This biosocial view contends, in other words, that cats and dogs likely feel some representation of affect and the motivations to (in)action it supplies, but do not experience emotions because they lack the capacities to be enculturated with the necessary concepts (Barrett 2017a). In this view, emotions are just as sociological as psychological or biological, and this “partnership” allows us to *share* our feelings and *collaborate* with one other in culturally elaborated and thus generative model-dependent (i.e., informative and predictable) ways. This view integrates much health physiology with affect via allostasis and interoception via cycles of action and perception in the body (*b* subscripts in Fig. 4). By adding the cultural cognitive superstructure of meaning-making by which actors can signal and share physiological regulation patterns with one another, emotions facilitate mutual understandings of the salience of situations, and scaffold decision-making (Massey 2002). Emotions thus allow an actor to communicate conceptually with oneself about their self, to share that knowledge with others, to interpret and make sense of others’ states, and to entrain with one another to reinforce social collectivity by sharing bodily and conceptual models of situations.

Emotions in Fig. 4 are therefore a nexus of body, situation, and culture. Quite subtly here, this implies a deep correspondence between emotions and cognition that undermines the common emotion–rationality dualism in much Western thought. Thinking and feeling are concurrent and intertwined with information on body states, and making meaning of body states is deeply intertwined with thinking. Indeed, our bodies are part of our generative models, and our actions are inevitably in service to them (Clark 2023; Mitchell 2023). Part of thinking is thus feeling, and the sense making of that emerges out of enculturation. Lizardo et al. (2016; see also Leschziner 2019) provides a detailed review of dual process theories of cognition in cultural sociology, emphasizing enculturation in terms of phases comprising paired “fast” and “slow processes”: cultural learning (cultural acquisition), remembering (storage of culture), thinking (processing of culture), and action (use of culture) (e.g., Vaisey 2008; Swidler 2008). Within the predictive Bayesian brain framework, learning, remembering, and thinking are integral components of the generative models that encode the statistical regularities observed inside and outside the body. These cognitive processes work in tandem, equipping actors with the diverse cognitive tools necessary for guiding perception and planning complex action policies, both toward concrete and abstract ends, and over arbitrary lengths of time.

*Learning* involves the processes by which generative models are made, elaborated, selected, and optimized through action–perception cycles and consequent predictive coding dynamics (Friston et al. 2016, 2017b). Actors become socialized and enculturated as they update and confirm their beliefs from experience to experience. *Remembering* provides for active reconstruction of past experiences, simulating prior sensory inputs and other forms of self-history, enabling deep and adaptive temporality (Badcock et al. 2019; Friston et al. 2018). *Thinking* is simulation with countless evaluative and projective uses, such as the formation of complex action policies projecting into the future. Thinking can lead to learning via the conscious evaluation of cognized prediction errors over different potential models, and thus can be conceived of as involving model development, state expansion, reduction, and selection (Friston et al. 2016; Ramstead et al. 2022; Sandved-Smith et al. 2021; Smith et al. 2020). Within this framework, learning updates or confirms beliefs, remembrances are beliefs about the past, and thinking combines the two and brings on-board beliefs about both alternative models and potential futures.

The process of enculturation plays a pivotal role in providing actors with the tools they need to predict the future states of their bodies and surroundings. This predictive ability relies on one’s understanding of their identities and positions within the world, their capacities to act, and the potential costs and benefits associated with their actions. *Action* is thus viewed as *action policy selection*, a form of Bayesian model averaging over policies such that those policies that lead to preferred outcomes have a greater impact on predictions (Friston et al. 2018, 2017a). The point of action is thus the realization of predicted states; hence, action–perception cycles can be recast in terms of enculturation as action-learning/memory/thinking cycles. Learning, remembering, and thinking thus arise from action just as they are used to guide action; hence, the *active inference* in AIF, and thus the intertemporal and cyclic nature of action and perception. Notably, the AIF model does not draw distinctions between fast and slow “Type 1” and “Type 2” cognitions in achieving these capacities that are of such interest in cognitive sociology. However, it does propose certain parallels. For example, maintaining a body and brain requires countless processes and mechanisms that are modulated by descending cascades of allostatic signals, usually taken to happen quickly (although shifts in the causal dynamics can take place over longer periods of time, as with Type II diabetes or the development of atherosclerosis). Of course, these are not *cognitive* processes.

For higher-order processes of those that lead to externally observable behaviors and are more typically of sociological concern, Friston et al. (2016) argue that the important distinction is whether or not current states are sufficient to specify an action or whether it is necessary to consider *uncertainty*, and thus *deliberate*. They propose instead a distinction based on belief-free (no uncertainty) and belief-based (uncertainty) states. Habits emerge naturally from goal-direct behavior within this distinction, raising the possibility that “fast” cognitions or habitual actions reflect those that deploy “automatically” because the model has high confidence, not because of a *specific “Type 1” neural system*. In the body and its core regulatory dynamics, these are constituted by evolutionary adaptation (Sterling 2015, 2020; Mitchell 2023). In the predictive brain, such expressions are taken to be implicit precisely because they are powerfully tuned predictions with very precise priors. In other words, many fast “cognitive” processes are indeed the least deliberative precisely because they embody the predictions that a brain has the most confidence in. Such predictions are metabolically efficient because deliberate thinking is more effortful (Parr et al. 2023), and thus metabolically expensive, when compared with actions with high degrees of situational success that can be implemented without deliberation. Such a view, for example, provides an alternative account of social schema (cast as Type 1 responses in prior work) (Boutyline and Soter 2021), as well as insights into everyday microsituational rituals and their breaches (Garfinkel 1967; Goffman 1967), in terms of highly precise priors.

## Conclusion

We presented an introduction to the concepts of predictive processing, the Bayesian brain, and variational (approximate) Bayesian inference through the lens of the AIF. Admittedly, this hierarchical predictive brain model, though drawing on findings from across the neurosciences, is an ongoing project. Although the neuroscience will no doubt continue to complicate, the details may not be of particular sociological interest if the global normative model is preserved. Part of the appeal lies in the fact that this perspective seems to resolve certain mind–body and biology–social dualities, while offering a more natural framework for human cognitive prospectivity and other capacities. Although there are many more sociological concerns^25^ that we would like to have addressed beyond our thematic “bread-crumb trails,” this way of thinking about brains, and action and perception via and within bodies, furnishes human actors that speak to neurological, biological, and sociological concerns in each area. This framework is level-interdependent and it intercorrelates actors via shared patterns of (co-)enculturation, emphasizing both generative processes of the body and the world, and actors’ subjective generative models by way of the expected and variational free energy minimizing action–perception cycles propelling minds through time.

## Footnotes

1 For examples of neuroscience work by sociologists, see Kalkhoff et al. (2020); Kiat et al. (2018c); Kiat and Cheadle (2017, 2018); Kiat et al. (2018a, b); Kiat et al. (2016, 2017); Melamed et al. (2017) and Schauenburg et al. (2019).

2 Like others who have sought incorporation of brains—or at least key dynamics subserving minds—theoretically into sociological inquiry (e.g., Boutyline and Soter 2021; Massey 2002; Turner 2007, 2020; Vaisey 2009), we recognize that how the products and consequences of such dynamics are conceived vary across levels of abstraction (Bericat 2016; Ma-Kellams 2014; Turner 2009).

3 The trend in neuroscience is generally away from circuit-based renditions and toward complex and integrated system representations (Avena-Koenigsberger et al. 2018; Barrett and Satpute 2013; Bilek et al. 2022; Farahani et al. 2019; Ficco et al. 2021; Hilgetag and Goulas 2020; Sporns and Betzel 2016). Even regions considered critical for basic capacities are in communication with huge swathes of the brain and so may enable by *integration* rather than producing by local calculations (Aliko et al. 2023; Cooper et al. 2023). If this is true, then in the coming years many circuits so far presumed to produce specific outcomes will be revised and recognized instead to integrate multimodal information from widely throughout the brain (Barrett and Simmons 2015; Hutchinson and Barrett 2019; Pessoa and Adolphs 2010).

4 There do not appear to be actual neural “fingerprints” in the brain for even the so-called basic emotions (Lindquist et al. 2012; Siegel et al. 2018; Wager et al. 2015), with the same outcomes produced over different configurations of network mechanisms (i.e., neural degeneracy and multiple realizability; Kamaleddin 2022; Strappini et al. 2020). In fact, a large-scale cross-cultural test of emotion models accentuated the universality of a three-dimensional model that evokes *affect* (Smith and Schneider 2009) rather than Kemper’s (1978), Turner’s (2007, 2020), or other traditional emotion models.

5 Although dual process model proponents in cognitive cultural sociology suggest that such models might not be controversial, Ignatow (2021) argues to the contrary in terms of neuroanatomy (Barrett and Simmons 2015; Hutchinson and Barrett 2019; Pessoa and Adolphs 2010) and following Melnikoff and Bargh (2018), who maintain that most dual-process model assumptions remain untested and that the framework lacks general support, contradicts well-established findings (see also Barrett et al. 2004), and is not internally coherent (for other criticisms in the behavioral economics literature see Grayot 2020).

6 Sociologists have shown an interest in “mirror neurons” (e.g., Summers-Effler et al. 2015), which fit naturally within the predictive coding framework (Kilner et al. 2007; Shipp et al. 2013).

7 The concept of hierarchy in the brain is actually interpreted in multiple ways that integrate through the embedding of connections in the spatial and topological architecture of the brain, interweaving into the structural and functional features of intricate activity patterns (Hilgetag and Goulas 2020).

8 The “brain in a vat” is a hypothetical scenario where a brain is isolated in a vat and fed artificial sensory information, raising philosophical questions about the reliability of sensory perceptions and the nature of reality. This scenario, which intends to challenge our ability to distinguish between genuine experiences and simulations, is a contemporary version of Descartesʼs evil demon. Of course, it seems to us, the scenario is best viewed as a restatement of the actual state of affairs.

9 When it comes to perception, it is perhaps better to think in terms of *fitness* rather than *veridicality*. Evolution does not appear to select on veridical perception but rather on perceptions that optimize fitness (Hoffman 2019; Prakash et al. 2021).

10 Our use of the *affordance* concept follows Veissière et al. (2020). See also Linson et al. (2018) and Ramstead et al. (2016), who also follow-up on Gibson (1979).

11 Bayes’ Theorem relates conditional probabilities, defined here in terms of hypotheses or beliefs *h* and data or observations *o*. This theorem shows how to calculate the inferential probability of hypotheses, *P*(*h*|*o*), given evidence as: $P\left(h|o\right)=\frac{P\left(h{,}o\right)}{P\left(o\right)}=\frac{P\left(h\right)P\left(o|h\right)}{P\left(o\right)}.$ *P*(*h*) is the prior distribution hypotheses, *P*(*o*|*h*) provides the sampling of the probabilities of observations given these hypotheses and is also called the likelihood. *P*(*h,o*) is joint distribution of observations and hypotheses, sometimes referred to as the *generative model*. The final term, *P*(*o*), is the marginal probability distributions of all potential observations over hypotheses, which cannot always be calculated. Bayesian updating allows $P\left(h\right)\rightarrow P\left(h|o\right)\rightarrow P\left(h\right)\rightarrow \ldots$ over time, experience by experience. For a technical introduction to Bayesian statistics from a social scientist’s perspective, see Gill (2002).

12 Note that the maximum likelihood function $L(\theta =h;y=o)$ familiar to quantitative sociologists is proportional to the Bayesian posterior $P\left(h|o\right)\propto L\left(h;o\right)$ when the priors are uniform (see Gill 2002, p. 33), reflecting an emphasis on hypotheses that make the observed data more plausible (i.e., sampling probabilities; see Clayton 2021). In contrast, *P*(*h*|*o*) provides an inference on how probable hypotheses are given what has been observed (i.e., posterior inferential probabilities), the quantity most relevant to actors going about the business of life, and the quantity we expect that most researchers would prefer to have on hand as well.

13 I.e., $$P\left(o\right)P\left(h|o\right)=P\left(h{,}o\right)=P\left(h\right)P\left(o|h\right)$$.

14 Technically, a Markov Blanket, a conditional independence structure proposed by Pearl (1998) with an essential role in many theoretical treatments of active inference in biology and complex systems (e.g., Bruineberg et al. 2018; Kirchhoff et al. 2018; Ramstead et al. 2018; Rubin et al. 2020).

15 The Kullback–Leibler [KL] divergence expresses the average differences between two distributions, an approximating variational density *Q*(*x*) and the true or exact distribution *P*(*x*): $D_{KL}\left[Q\left(x\right)||P\left(x\right)\right]=\sum _{x\in X}P\left(x\right)\left[\ln Q\left(x\right)-\ln P\left(x\right)\right]$ (Smith et al. 2022; Parr et al. 2022), where $\bar{X}=\sum _{x\in X}P\left(x\right)x$.

16 A Markov process describes a sequence of states where the probability of each state depends only on the immediately preceding one. In this case, the past is reflected in current and changing neural structure (e.g., models and memories) and body states (e.g., health).

17 In Fig. 3, action policies are denoted with *π* and preferences with *C*.

18 These concepts are sometimes referred to as the *exploration* and *exploitation *dilemma (Berger-Tal et al. 2014), which this expected free-energy formulation resolves (see Parr et al. 2022).

19 Strand and Lizardo (2022b) describe how Weber’s later works were omitted from important English-language translations, while also providing additional references on the origins and implications of Weber’s probabilism.

20 There was great interest in probability and other leading intellectuals such as Kant (Swanson 2016) and Helmholtz (Friston and Kiebel 2009; see also the Appendix discussion in Hutchinson and Barrett 2019) advocated for predictive formulations. Debates about the definitions of probability have largely settled into “frequentist” (objective) and “Bayesian” (objective and subjective) camps (Clayton 2021), but were much more variable during Weber’s time.

21 It may be tempting to think about this as limited to basic perceptual experiences, but we should be careful to not overly constrain the abstraction. The intended meanings of this text are hidden states that you are attempting to infer. The chances that your inferences are accurate are undoubtedly a function of the clarity of writing, as well as what you bring to the table by virtue of your prior knowledge and capacities.

22 The paramount challenges of our era and into the foreseeable future include the escalating threats of global warming and the interdependent precarious erosion of biodiversity. Both reflect the consequences of actions that have changed the world to suit a broader range of our intrinsic and enculturated preferences and goals.

23 Medical sociologists have been greatly interested in depression and mental health over the years, and it is worth noting that there is considerable interest in depression as a problem of allostasis and/or interoception (e.g., Arnaldo et al. 2022; Barrett et al. 2016; Harshaw 2015; Seth and Friston 2016; Shaffer et al. 2022; Stephan et al. 2016).

24 Pulvermüller (2023) provides insights into neural dynamics by which language directs attention to conceptual features and symbols in the development of abstract concepts.

25 We have alluded to, but not strictly developed, the mathematical frameworks of the AIF. A deeper investigation has the potential to enable specifying neurobiologically realistic agents, creating exciting new avenues within theoretical computational sociology (see Foster 2018). A useful starting place can be found in Smith et al. (2022).
